# Subjective long-term emotional and social effects of recreational MDMA use: the role of setting and intentions

**DOI:** 10.1038/s41598-024-51355-6

**Published:** 2024-02-10

**Authors:** Timon Elmer, Tanya K. Vannoy, Erich Studerus, Sonja Lyubomirsky

**Affiliations:** 1 Applied Social and Health Psychology, Department of Psychology, University of Zurich, Zurich, Switzerland; 2 Department of Psychology, University of California, Riverside, USA; 3 Department of Psychology, University of Basel, Basel, Switzerland

**Keywords:** Psychology, Human behaviour

## Abstract

MDMA is a recreational drug commonly used to enhance euphoria, but it is also used in non-party settings with self-insight or social connection intentions. Yet, little is known about whether distinct consumer groups are formed based on consumption setting and intention. We aimed to characterize different types of recreational MDMA users based on consumption setting and intentions, and to examine their differences in perceptions of long-term social-emotional effects of MDMA use. We analyzed self-reports of 766 individuals (ages 18–61, mostly from Western countries), reporting on their MDMA consumption habits and perceived effects. We used a K-medoids clustering algorithm to identify distinct types of consumption settings and intentions. We identified three setting types – party settings with friends (N = 388), private home settings (*N* = 132), mixed settings (*N* = 246) – and three intention types – euphoria and energy (*N* = 302), self-insight (*N* = 219), mixed intentions (*N* = 245). Members of the self-insight and mixed intentions clusters reported considerably more long-term socio-emotional benefits than members of the euphoria and energy cluster. No differences were observed between the setting clusters. In this particular sample, more long-term benefits than harms were reported. Our findings suggest that the long-term social-emotional benefits of MDMA are associated with whether users seek self-insight or have mixed intentions.

## Introduction

MDMA, also known as "ecstasy" or "molly," is the chemical compound ( ±) 3,4-methylenedioxy-methamphetamine. It is a psychoactive drug that has gained increasing popularity as a recreational substance. In the UK, for example, the lifetime prevalence is at almost 10% and in the EU it is estimated that 1.4% of young adults between the ages of 15 and 34 have consumed MDMA in the past year^1^. Its short-term effects, such as feelings of euphoria, reduced fear and negativity, and increased sociability, have been well-documented^2–7^.

Yet, to date, less is known about the long-term social and emotional benefits of recreational MDMA use. Past research has primarily focused on negative cognitive and emotional effects e.g.^8–11^ or has highlighted positive effects mainly in therapeutic settings^12–14^. Although some research suggests that recreational MDMA use may also have positive long-term effects^15,16^, much remains to be learned about what factors contribute to positive emotional and social outcomes in the long-term. Johnstad^17^ recently addressed this research gap by pointing out that “[…] we currently have little knowledge about how psychedelics [such as the semi-psychedelic MDMA] are used in naturalistic settings in Western societies. We recognize that there is a range of different patterns of use, some of which clearly have better long-term outcomes than others, but we have not obtained much insight into how widespread these patterns are” (p. 46). The present study aims to address this gap by investigating patterns of recreational MDMA consumption and their potential long-term benefits and harms in terms of emotional experiences and feelings of social connection.

The set and setting hypothesis^18,19^ helps to explain why some patterns of MDMA use are linked to more positive experiences than others. This hypothesis suggests that the subjective effects of psychoactive substances, such as MDMA, are influenced by both individual and environmental factors. Variables such as the user's personality, mood, preparation, expectation, and intention—summarized as the *set* factors—as well as the physical, social, and cultural context in which the substance is consumed—collectively referred to as the *setting* factors—can significantly influence the nature and evaluation of the experience with a psychoactive substance^18^. Previous research has shown that individual characteristics, such as sex^20–22^, personality^23^, prior drug experience^24,25^, and social context^26^ can predict the short-term (i.e. acute) effects of MDMA use. However, limited research exists on the influence of set and setting on the long-term effects of MDMA use.

In studying the patterns of recreational MDMA use and its long-term effects, we specifically focus on (a) the physical and social settings in which individuals typically consume MDMA and (b) the intentions with which MDMA is consumed. With regards to settings, it is stereotypically assumed that most recreational MDMA use occurs at parties with friends^27,28^. However, different types of recreational MDMA users may have different preferences for the setting in which they consume the drug, such as in private homes or out in nature^16,27, 28^. To date, little research has examined the types of recreational MDMA users and their preferred settings.

Another important factor that may influence the long-term effects of MDMA use is the user's intention to take the substance^13^. Intentions, which are part of the *set* component of the set and setting hypothesis, refer to the motivations and expectations of using a psychoactive drug^18^. As such, the intention with which individuals consume MDMA may differ widely across individuals. Some people might take MDMA to feel energized, euphoric, and social, while others might take it to obtain self-insight^7,28, 29^. To date, however, no study has identified prototypical types of recreational MDMA consumers with regard to their intentions. If the set and setting hypothesis holds ground, and people who consume psychedelic-like substances in more favorable sets and settings have better experiences on a variety of dimensions, then these positive short-term experiences may cumulate into more favorable experiences in the long run.

### The current study

The current study aims to address these gaps in the literature by, first, examining different types of recreational MDMA users and their preferred settings and commonly-held intentions. Second, we examine the relationship between the identified types of MDMA users and their self-reports of the personal long-term socio-emotional effects of MDMA use. Specifically, we will address the following three research questions:

1. Which types of recreational MDMA users exist with regards to the settings in which they experience MDMA?
2. Which types of recreational MDMA users exist with regards to the intentions with which they consume MDMA?
3. Are there differences in the perceived long-term social-emotional effects of MDMA use between the identified types of MDMA consumers (in terms of settings and intentions)?

We explored these three research questions using a dataset collected by Elsey et al.^30^, who surveyed 886 recreational MDMA consumers about the perceived short- and long-term benefits and harms they subjectively experienced after consuming MDMA. Elsey et al.^30^ collected the data with the intention of providing an openly available dataset on the long-term effects of recreational MDMA use. Although their preprint provides various descriptive aspects of the data, including bivariate correlations between the variables collected, they do not differentiate between types of MDMA consumers and how they differentially perceive these long-term social and emotional outcomes. Moreover, in our analyses, we investigate these associations not only bivariately, but using a multivariable regression framework—allowing us to control for potentially confounding variables such as age, gender, frequency of MDMA use, MDMA addiction, and mental health conditions. By understanding the factors that may influence the long-term effects of MDMA use, this study aims to add to the gaps in the literature and help inform safe consumption that promotes positive long-term socio-emotional outcomes.

## Results

### RQ1: which types of recreational MDMA users exist with regards to the settings in which they experience MDMA?

Sample averages of the physical and social setting variables (RQ1) and intention variables (RQ2) are presented in Table S3 of the Supplementary Materials. An analysis of the eigenvalues of the setting variables revealed the presence of four eigenvalues above 1 (3.03, 1.76, 1.31, 1.13, 0.98, …). Thus, the first four principal components of the settings variable were used for the cluster analysis. To determine the ideal number of clusters, the Medoids clustering algorithm was applied on *k* numbers of clusters and the value of *k* with the highest average silhouette width *S* was chosen, as it is a measure of how well data points are assigned to clusters^31^. Our analysis yielded the following values: *S*_*k*=*2*_ = 0.31, *S*_*k*=*3*_ = 0.34, *S*_*k*=*4*_ = 0.32, *S*_*k*=*5*_ = 0.30, *S*_*k*=*6*_ = 0.25. Given that *k* = 3 showed the highest S value, our participants were assigned to three different clusters with sizes *N*_*1*_ = 388, *N*_*2*_ = 132, *N*_*3*_ = 246. For robustness analyses on alternative clustering analysis choices with (a) *k* = 2 and (b) clustering analysis without prior PCA, please refer to the Supplementary Materials Figs. S1–S4.

**Figure 1:**
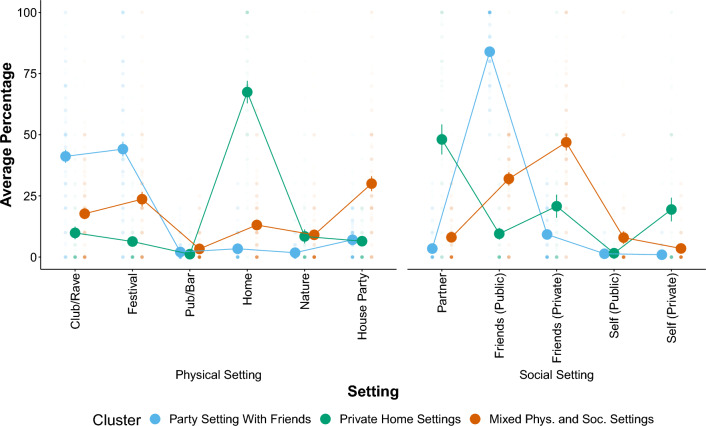
Three identified setting clusters and their average values per setting variable. Large dots with error bars represent averages and 95% confidence intervals. Smaller transparent dots represent raw values. Some confidence intervals are so small that they are invisible in this figure.

Figure 1 illustrates the three identified clusters and their average values per setting variable. We named the three setting clusters according to their unique patterns displayed in Fig. 1. The first cluster was named Party Setting With Friends, characterized by consuming MDMA in clubs, raves, or festivals together with friends. The second cluster was named Private Home Settings, as individuals in this cluster mostly consume MDMA at home with their partner, friends, or by themselves. The third cluster was named Mixed Physical and Social Settings, as individuals in this cluster consume MDMA in various physical and social settings.

Tables S7 and S8 in the Supplementary Materials report the results of analyses of differences between the setting clusters in terms of a number of covariates (i.e. age, gender, frequency and recency of MDMA use, CAGE MDMA addiction score, mental health status). There were no differences between the setting clusters in terms of frequency and recency of MDMA use (*F*_*frequency*_(2) = 1.062, *p* = 0.346; *F*_*recency*_(2) = 1.727, *p* = 0.178), and mental health status (χ^2^ (4) = 4.31, *p* = 0.120), but there were significant differences between the setting clusters in the CAGE MDMA addiction score (*F*_*setting*_(2) = 6.23, *p* = 0.002), age (*F*_*setting*_(2) = 31.59, *p* =  < 0.001), and gender identity (χ^2^ (4) = 13.32, *p* = 0.010). CAGE MDMA addiction scores were highest in the Mixed Setting cluster with *M* = 1.35 (*SD* = 1.18), whereas the Party Setting with Friends cluster scored on average 1.15 (*SD* = 1.18) and the private home setting *M* = 0.90 (*SD* = 1.17). Participants in the cluster Party Setting with Friends were on average 27.34 (*SD* = 7.26) years old, whereas the private home setting cluster and the mixed setting clusters were on average 34.30 (*SD* = 11.37) and 30.01 (*SD* = 9.46) years old, respectively. There were more females than males in the Party Settings with Friends cluster (*N*_female_ = 211; *N*_male_ = 173; *N*_other_ = 4) and slightly more males in the mixed setting cluster (*N*_female_ = 113; *N*_male_ = 121; *N*_other_ = 12).

### RQ2: Which types of recreational MDMA users exist with regards to the intentions with which they consume MDMA?

Following the analysis of RQ1, we proceeded to examine the eigenvalues of the intention variables, revealing two eigenvalues above one: 2.27 and 1.44, respectively. We then utilized the two principal components of a PCA on the intention variables. Subsequently, the Medoids clustering algorithm was executed on *k* numbers of clusters to determine the *k* with the highest average silhouette width *S* for further analysis. The resulting values were as follows: *S*_*k*=*2*_ = 0.35, *S*_*k*=*3*_ = 0.40, *S*_*k*=*4*_ = 0.33, *S*_*k*=*5*_ = 0.34, *S*_*k*=*6*_ = 0.34. Based on these results, we assigned our participants to three distinct clusters, with *k* = 3 having the highest *S* value. The three clusters were of sizes *N*_*1*_ = 302, *N*_*2*_ = 219, *N*_*3*_ = 245. For robustness analyses with alternative clustering analysis choices (a) *k* = 2 and (b) clustering analysis without prior PCA, please refer to Supplementary Materials Figs. S1–S4.

**Figure 2:**
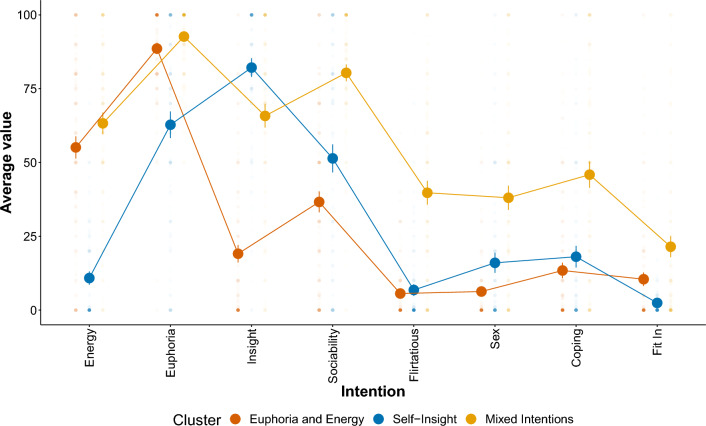
Three identified intention clusters and their average values per variable. Large dots with error bars represent averages and 95% confidence intervals. Smaller transparent dots represent raw values. Some confidence intervals are so small that they are invisible in this figure.

In Fig. 2, the assignment of individuals to the three identified clusters is displayed along the population’s average values per intention variable. The clusters were named according to the observed patterns in Fig. 2. The first cluster was dubbed Euphoria and Energy due to the high average values on the euphoria and energy items, and relatively low values on all other items. The second cluster, which we named Self-Insight, consists of individuals scoring high mainly on the insight item. The third cluster was composed of individuals with Mixed Intentions, scoring higher on most items compared to the other two clusters of individuals.

As described in Tables S7 and S8 in the Supplementary Materials, there were no differences between the intention clusters in terms of gender identification (χ^2^ (4) = 3.01, *p* = 0.556) and frequency and recency of MDMA use (*F*_*frequency*_(2) = 0.06, *p* = 0.801; *F*_*recency*_(2) = 2.35, *p* = 0.126), but differences in terms of age (*F*(2) = 13.89, *p* =  < 0.001), mental health status (χ^2^ (4) = 17.26, *p* =  < 0.001), and CAGE MDMA addiction score (*F*(2) = 12.68, *p* =  < 0.001). Individuals in the Self-Insight cluster were on average 31.75 years old (*SD* = 10.13), whereas the mixed intention cluster was *M* = 29.95 (*SD* = 8.95) years old. The Euphoria and energy cluster was the youngest with *M* = 27.24 years old (*SD* = 8.06). The Euphoria and Energy cluster consisted of fewer participants (25.2%), who reported ever having had a mental health diagnosis, compared to the self-insight (38.8%) and mixed intentions clusters (40%). The average CAGE MDMA addiction score was highest in the mixed intention cluster (*M* = 1.54, *SD* = 1.24). The Self-Insight *(M* = 0.78, *SD* = 1.02) and Euphoria and energy clusters (*M* = 1.15, *SD* = 1.16) scored lower on average.

Table 1 shows the overlap between the setting and intention clusters. Most notable is the strong overlap between individuals in the Party Setting with Friends cluster and the Euphoria and Energy intention cluster: 55% of the individuals assigned the Party Setting with Friends cluster were also in the Euphoria and Energy intention cluster. Those individuals consuming MDMA mostly in Private Home Settings were, in 61% of the cases, also part of the self-insight intention cluster. The Mixed Physical and Social Setting cluster was about equally represented in all three intention clusters.

**Table 1:** Cross-table between setting and intention clusters.

	Euphoria and energy	Self-insight	Mixed intentions	Total
Party setting with friends	212	57	119	388
Private home settings	14	81	37	132
Mixed phys. and soc. settings	76	81	89	246
Total	302	219	245	

### RQ3: are there differences in the perceived long-term social-emotional effects of MDMA use between the identified types of MDMA consumers (in terms of settings and intentions)?

Participants generally reported high levels of perceived benefits (*M* = 55.2; *SD* = 36.9) and low levels of perceived harms (*M* = 8.63; *SD* = 19.86), as displayed in detail in Table S4 of the Supplementary Materials. Table 2 presents the means per identified setting and intention cluster of the long-term social-emotional variables. Additionally, the results of an ANOVA analysis, aimed at examining differences in means of long-term variables between the clusters, are also displayed in Table 2. The findings indicate that only a few long-term variables show significant differences between the setting clusters, whereas most long-term variables show significant differences exist between the intention clusters. To further examine these patterns while accounting for relevant covariates (see “Methods” section), we conducted a series of multivariate linear regression models.

**Table 2:** Mean (and SD) of perceived long-term benefits and harms by setting and intention clusters.

	Setting clusters								Intention clusters							
	Party setting with friends		Private home settings		Mixed settings		*F*	*p*	Euphoria and energy		Self-insight		Mixed intentions		*F*	*p*
	*M*	*SD*	*M*	*SD*	*M*	*SD*			*M*	*SD*	*M*	*SD*	*M*	*SD*		
Best experience ever	70.4	28.9	80.8	25.1	73.1	29.5	6.47	0.002	63.1	31.0	78.7	26.6	80.3	23.8	32.57	< 0.001
Amazing memories	86.1	21.8	85.1	21.8	86.3	23.0	0.14	0.869	84.5	23.2	86.3	22.3	87.5	20.6	1.29	0.275
Appreciate aesthetic experiences	58.4	34.3	63.9	32.9	62.2	34.6	1.69	0.186	47.7	34.9	70.8	30.5	67.3	31.4	39.71	< 0.001
Improved friendship quality	57.8	36.0	61.1	34.5	66.8	33.2	4.98	0.007	48.9	36.8	70.2	30.8	68.5	31.9	33.83	< 0.001
Increased social confidence	41.7	33.2	52.5	35.4	50.9	33.8	8.05	< 0.001	31.1	31.7	57.7	32.3	55.6	31.4	58.98	< 0.001
Increased positivity	45.7	37.0	71.4	31.9	56.6	36.7	26.11	< 0.001	31.9	34.1	74.6	29.6	61.6	33.2	119.80	< 0.001
Increased authenticity	53.9	34.7	64.8	32.9	59.6	34.1	5.60	0.004	42.8	34.9	70.0	29.9	64.8	31.0	53.91	< 0.001
Increased compassion	43.3	36.2	65.2	32.9	55.2	36.1	21.20	< 0.001	30.1	32.6	69.3	31.4	60.1	32.8	107.64	< 0.001
Better emotion reflection	47.2	36.6	66.8	31.8	57.9	34.4	17.66	< 0.001	33.8	33.5	72.7	29.8	62.2	31.1	107.51	< 0.001
Deep emotion experience	44.2	36.0	64.0	35.0	55.0	35.6	17.33	< 0.001	32.7	34.2	69.0	32.7	57.7	32.0	83.79	< 0.001
Positive world view	45.5	33.5	60.7	35.5	51.6	32.7	10.38	< 0.001	34.2	31.1	66.5	31.4	55.1	31.2	72.57	< 0.001
Reduced other drug use	16.1	28.0	24.0	34.0	17.6	29.7	3.51	0.030	11.0	24.1	25.7	34.7	19.5	29.4	16.79	< 0.001
Worst experience ever	11.3	23.5	9.1	19.1	12.9	25.6	1.13	0.322	11.2	23.5	7.1	16.1	15.7	28.0	7.94	< 0.001
Unpleasant memories	10.0	20.0	7.7	16.5	9.4	21.6	0.70	0.497	8.4	18.7	5.2	14.8	14.4	24.1	13.25	< 0.001
Dampen aesthetic experiences	8.0	18.7	6.4	17.2	7.6	18.6	0.39	0.679	8.4	19.6	2.9	10.1	10.9	21.4	11.84	< 0.001
Worsened friendship quality	3.8	12.9	4.6	12.5	5.0	15.3	0.60	0.552	3.0	11.7	2.3	8.7	7.7	18.2	11.58	< 0.001
Increased social anxiety	8.4	19.1	4.9	15.9	7.5	18.4	1.77	0.172	5.5	14.8	4.5	14.2	12.8	23.8	15.50	< 0.001
Decreased positivity	17.3	29.0	12.3	26.7	16.9	28.5	1.57	0.208	16.6	29.7	8.7	21.0	22.7	31.2	14.40	< 0.001
Increased paranoia	6.6	17.6	4.4	13.3	5.6	14.5	1.01	0.364	5.7	16.6	2.0	5.9	9.7	19.9	13.75	< 0.001
Issues concentrating	15.5	24.2	12.4	24.9	16.2	24.8	1.10	0.334	14.9	24.7	8.6	18.0	21.5	27.6	16.66	< 0.001
Worse emotion reflection	5.5	14.4	3.5	13.9	6.6	17.9	1.74	0.176	5.2	14.7	2.0	7.9	9.1	20.3	12.39	< 0.001
Shallow emotion experience	7.5	15.6	6.6	19.0	8.7	19.0	0.70	0.497	7.2	16.0	2.9	10.3	12.8	22.0	19.94	< 0.001
Negative world view	4.6	12.1	4.7	16.3	4.9	13.9	0.05	0.950	3.3	10.1	2.3	7.8	8.6	19.1	16.15	< 0.001
Increased other drug use	7.4	19.0	8.2	20.1	8.5	19.0	0.28	0.757	5.3	14.7	3.9	13.0	14.6	25.9	24.01	< 0.001

For each benefit and harm item, participants were asked how MDMA may have affected them over the long term (i.e. at times when they are not on MDMA or recovering from taking MDMA). *N* = 766, *df* = 2, *M* mean, *SD* standard deviation, *F* = F value of an ANOVA analysis comparing the cluster mean.

**Figure 3:**
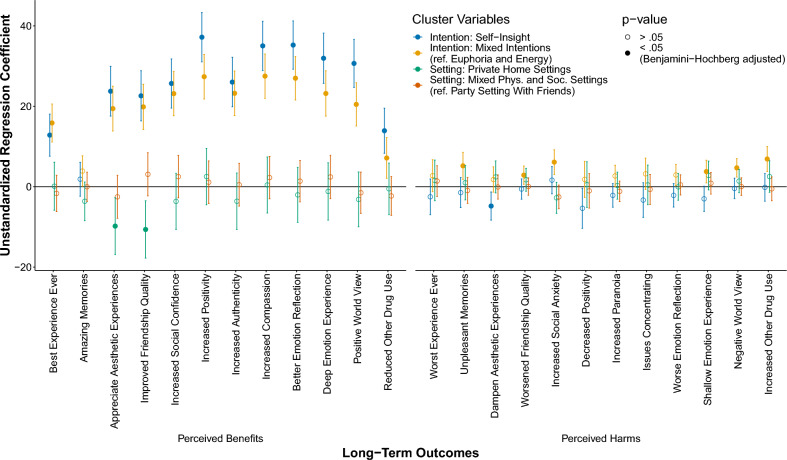
Unstandardized regression coefficients of the setting and intention cluster variables for each perceived long-term benefit and harm. Unstandardized regression coefficients include error bars representing 95% confidence intervals. For the two intention-dummy variables (Self-Insight, Mixed Intentions), the reference category is the cluster Euphoria and Energy. For the two setting-dummy variables (Private Home Setting, Mixed Setting), the reference category is the cluster Party Setting with Friends. Empty dots represent coefficients with Benjamini–Hochberg adjusted *p*-values that did not pass the α threshold of 0.05. Full dots indicate coefficients that were below the (Benjamini–Hochberg adjusted) *p*-value of 0.05. Control variables: Gender, age, the frequency of MDMA use within the past 6 months, the number of months since the last MDMA use (with a maximum value of 24), CAGE MDMA addiction score, and whether the participant reported having been diagnosed with a mental health problem.

Figure 3 shows the unstandardized regression coefficients for each long-term variable separated by the setting and intention cluster variables. The full model results can be found in the R-Markdown file at OSF https://osf.io/t7p59.

#### Setting clusters

As shown in Fig. 3, the results indicate that the two setting variables—Private Home Settings and Mixed Physical and Social Settings—did not differ significantly in any of the perceived positive or negative long-term variables from the cluster Party Setting With friends. Only the Private Home Setting cluster showed slightly lower values in appreciate aesthetic experiences ${(b}_{setting = 2} =-9.79,t\left(754\right)= -2.72, p = .023)$ and improved friendship quality ${(b}_{setting = 2} =-10.63,t\left(754\right)= -2.93, p = .013)$ than the Party Setting With Friends cluster.

#### Intention clusters

By contrast, the two intention clusters—Self-Insight and Mixed Intentions—differed significantly from the Euphoria and Energy cluster in most positive long-term effect variables (see left panel of Fig. 3). These effect sizes—with an average standardized *β* of 0.30 (*SD* = 0.11), ranging between 0.04 and 0.45—were generally medium to large^32^ – only for the Amazing Memories variable did these clusters not differ (${b}_{intention = 2} =1.87,t(754) = 0.87, p = .595$, ${b}_{intention = 3} =3.90,t(754) = 2.01, p =0 .126$). To contextualize these effect sizes, we examine the association of belonging to the Self-Insight cluster on the perceived long-term positive effects on friendships, indicated by β = 0.29 (i.e. the average effect size). In our model, being in this cluster, as opposed to the Euphoria and Energy cluster, is linked to a substantial increase of 23 points on a scale of 0–100. This reflects a notably large effect.

Interestingly, the two intention clusters, Self-Insight and Mixed Intentions, did not differ significantly from the Euphoria and Energy cluster in most negative long-term effect variables (see right panel of Fig. 3). Only the Mixed Intentions cluster differed from the Euphoria and Energy cluster in unpleasant memories (${b}_{intention = 3} =5.12,t(754) =3.02, p = .010$), worsened friendship quality (${b}_{intention = 3} =2.89,t(754) =2.49, p = .040$), increased social anxiety $({b}_{intention = 3} =6.12,t(754) =3.91, p <.001$), shallow emotion experiences (${b}_{intention = 3} =3.77,t(754) =2.61, p = .029$), negative world view $({b}_{intention = 3} =4.70,t(754) =4.02, p <.001$), and increased other drug use ${(b}_{intention = 3} =6.90,t(754) =4.36, p <.001$). The Self-Insight cluster showed lower values in the dampened aesthetic experiences variable compared to the Euphoria and Energy cluster (${b}_{intention = 2} =-4.80,t\left(754\right)=-2.67, p = 0.024$).

#### Explained variance

**Figure 4:**
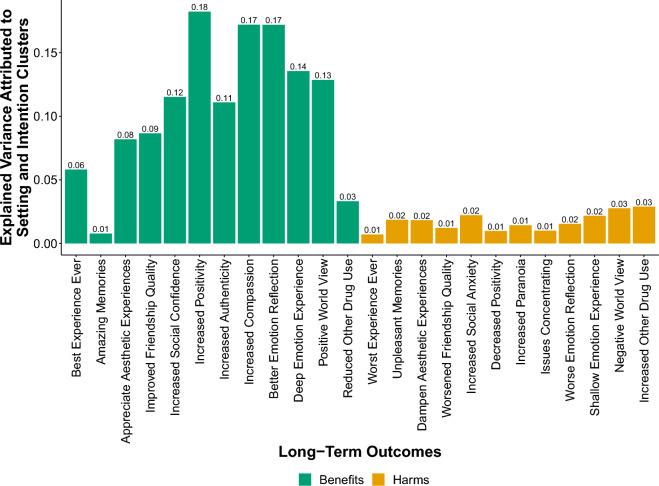
Explained variance attributed to the setting and intention cluster variables per long-term outcome.

By comparing the explained variance R^2^ of linear regression models with and without the setting and intention clusters (while controlling for covariates), we further assessed how much of the explained variance of the outcome variables can be attributed to the setting and intention clusters. Figure 4 shows the explained variance R^2^ attributed to the setting and intention cluster variables per long-term outcome variable. Setting and intention cluster variables explained a notable proportion of the variance in long-term benefits with an average of (*M* = 0.11, *SD* = 0.06), but not for the long-term harms (*M* = 0.02, *SD* = 0.01).

### Robustness analyses

We conducted several analyses to test the robustness of our results under differ assumptions and procedures. Specifically, (a) we assessed a two-cluster solution for the setting and intention variables, (b) we applied the clustering algorithms without performing a PCA first, (c) we analyzed RQ3 with the principal components as independent variables instead of the clusters identified by the clustering algorithm, and (d) we ran a regression analysis without any covariates (i.e. only the dummy variables for the intention and setting clusters). The conclusions drawn from these robustness analyses are identical to the ones described in this Results section. For details on the results of these robustness analyses, please see the section Robustness Analyses in the Supplementary Materials.

## Discussion

This study aimed to identify different types of recreational MDMA users with regards to the setting (RQ1) and intentions (RQ2) of their MDMA consumption, and to investigate whether these types of users report systematically different long-term social-emotional benefits and harms (RQ3). Our analyses identified three distinct clusters of recreational MDMA users based on the physical and social settings in which they typically consume MDMA: Party Setting With Friends, Private Home Settings, and Mixed Settings. We further identified three distinct clusters based on users’ intentions for consuming MDMA: Euphoria and Energy, Self-Insight, and Mixed Intentions. These findings are consistent with previous research that has identified a variety of typical settings in which MDMA is consumed, with a large proportion of individuals consuming MDMA in physical settings such as nightclubs, outdoor festivals, or private homes and with hedonic, introspective, and social motives^27,28^.

Regarding the long-term social and emotional outcomes, participants generally reported high levels of benefits and very low levels of perceived harm. Our results further indicate that the intentions participants described were strongly associated with the long-term social-emotional benefits they reported, but not with the harms. Specifically, individuals in the Self-Insight and Mixed Intentions clusters reported proportionally higher long-term benefits attributed to MDMA consumption, such as improved social relationships, empathy, and emotional experiences, compared to individuals in the Euphoria and Energy intention cluster. With an average standardized *β* of 0.30 (SD = 0.11), these effect sizes were generally medium to large^32^. By contrast, the physical and social settings in which participants consumed MDMA were not associated with perceived long-term social-emotional benefits or harms. These findings indicate that consuming MDMA with particular intentions (e.g. using MDMA to gain self-insight) rather than consuming it in particular settings may be associated with relatively more beneficial social and emotional experiences in the long-term.

Overall, these findings suggest that recreational MDMA users have different motivations and intentions when consuming this substance. Long-term social-emotional effects, following MDMA consumption, also differ among these distinct groups. These results provide support for growing literature emphasizing the importance of considering the complexity of MDMA consumption, instead of oversimplifying it as either positive or negative^11,15, 33, 34^.

Our findings also suggest that individuals who use MDMA with the intention of gaining self-insight or for mixed purposes may experience more favorable long-term outcomes, such as enhanced social functioning and emotional experiences, compared to those who primarily use it for hedonistic reasons (i.e., to feel euphoric or energetic). This information could be used to inform harm reduction strategies for MDMA use, as well as to help individuals make informed decisions about their use of psychoactive substances. For example, interventions aimed at individuals in the Euphoria and Energy cluster could focus on improving their experiences through psycho-education about the importance of set and setting factors. Although the setting clusters did not differ in long-term outcomes, they may help to provide information on where such interventions could be effective: The individuals who consumed MDMA mainly with Euphoria and Energy intentions—associated with less positive long-term effects—mainly consumed MDMA in a Party Setting With Friends (see Supplementary Materials Table S6 for details). Existing programs to inform and care for MDMA consumers at festivals and parties may therefore represent effective locations for such psycho-education interventions.

These findings add to our understanding of the complex interplay between MDMA consumption, set and setting factors, and social-emotional functioning. The importance of intentions has been emphasized in therapeutic contexts (e.g. Ref.^35^), and this study suggests that one’s intentions may play a crucial role in perceived long-term psychosocial benefits. According to Greer and Tolbert^13^, it is important that MDMA is used with the explicit goal of learning from the experience: “Taking MDMA with an intention to learn, with an attitude of acceptance, and in a safely structured setting enabled people to experience their true nature which is essentially loving and forgiving” (p. 34). The importance of intentions has also been emphasized by findings of Phase III clinical trials on applying MDMA together with (self-insight focused) pre- and post-session psychotherapy to treat PTSD. In the latest trial, 67% of MDMA recipients no longer met PTSD diagnosis criteria after the 18-week trial^36,37^.

Unique about this study is its depiction of how people typically consume MDMA across a variety of settings and with various intentions. Johnstad^17^ argued that the research community needs better knowledge about these contextual factors in order to better understand the consumption of psychedelics and related substances in (Western) societies. Learning about typical consumption patterns across a variety of settings helps researchers to know when (not) to generalize across different types of MDMA users. For example, studies that recruit participants at a festival or rave (e.g. Ref.^38^ ) may end up not including individuals who consume MDMA mainly in private settings.

The findings of this study align well with the existing findings on the perceived benefits of recreational consumers of classic psychedelics (e.g. LSD, psilocybin, mescaline, ayahuasca; excluding MDMA). For example, Wolff et al.^39^ found that individuals with high levels of therapeutic intentions are likely to show shifts in psychological flexibility (which is argued to be a key factor for therapeutic success), whereas individuals with high levels of hedonic intentions or escapist intentions do not^39^. Similarly, Haijen et al.^41^ showed that having clear intentions when consuming the classic psychedelics was associated with more mystical-type experiences, which in turn, was related to more positive long-term effects on well-being.

Finally, it is important to consider this study’s limitations. First, the study may have oversampled individuals who had relatively more positive experiences with MDMA, which could have biased the results towards more positive outcomes. For this reason, our findings should be interpreted cautiously, as they are not generalizable to wider populations. Future research should prioritize the collection of a more representative sample of the diverse population engaging in MDMA use to enhance the external validity of research findings. This approach would provide a more comprehensive understanding of the varied experiences associated with MDMA consumption across different user profiles.

Second, unobserved confounding variables might have also biased our results. For example, individuals who report generally higher happiness may be more likely to consume MDMA for insight and also to see it as a more positive experience. At the same time, it is possible that individuals who already believe in the potential positive effects of MDMA are more likely to report using it for self-insight, and to perceive positive changes as a result. Furthermore, it is conceivable that individuals who have experienced positive changes from MDMA use in the past subsequently use it for the purpose of inducing yet more positive changes. Finally, it may be that individuals who consume MDMA in private settings for self-insight may do so less frequently than those who consume it in party settings for euphoria and energy (although we observed no significant differences in frequency of MDMA use between cluster variables, see Supplementary Materials Table S7). By controlling for a number of important and potential confounding factors (age, gender, frequency of MDMA use, MDMA addiction, and mental health conditions), we aimed to reduce the effects of such confounding factors.

Third, this study relied solely on self-reported measures of setting, intention, and long-term effects, which may be subject to memory biases, schemas, stereotypes, and preconceptions. The cross-sectional design of the study also precludes any causal inferences about the relationship between MDMA use and long-term outcomes: It may be that participants are biased towards (wrongly) attributing perceived social-emotional developments to their MDMA experiences, while misattributing harms to external factors. Future work using different methodologies (e.g. longitudinal) should be considered to assess the replicability of these findings, given this is the first study that assesses long-term benefits/harms based on setting and intentions.

A fourth limitation is that the study did not measure how many times participants had used MDMA, which could have affected their categorization into different clusters. However, our proxy for the overall use of MDMA –MDMA consumption over the past 6 months – did not moderate the associations between setting/intention and long-term outcomes (see R-Markdown analysis on https://osf.io/t7p59). Hence, these data suggest no additional differences between the clusters for individuals who consumed more MDMA within the past 6 months compared to those who consumed less MDMA in the past 6 months.

One potential avenue for future research is to develop interventions that aim at changing people’s intentions for consuming MDMA and test whether such interventions produce positive long-term social-emotional effects. Additionally, future investigators could examine the potential moderating factors that may influence the long-term effects of MDMA use, such as its dosage, frequency of use, and other features of set and setting. Moreover, it would be worthwhile to explore the mechanisms underlying the differences in long-term effects between different types of recreational MDMA users. For example, people who have insight intentions may have a higher need for integration after the experience, which leads to more positive long-term effects. Finally, this study did not find any meaningful effects on long-term outcomes based on the type of setting in which MDMA is consumed. The role of setting in influencing long term socio-emotional effects following MDMA use requires closer investigation. Future research could explore whether setting plays a role in individuals’ comfort levels and sense of control in the setting rather than the setting itself.

## Conclusion

“We know that some people take psychedelics infrequently in carefully planned sessions for spiritual, therapeutic and developmental reasons^40^, while others perhaps use psychedelics very frequently for entertainment or escapist purposes, and we should not be surprised if these usage patterns are associated with very different long-term consequences.” (Johnstad, 2021, p. 36).

While much research has examined the short-term socio-emotional effects of MDMA administration (during the experience or in the days thereafter), as well as the long-term effects and benefits of *classic* psychedelics in recreational consumers^41^, e.g. Refs.^42,43^, little work has been devoted to the long-term socio-emotional effects of MDMA consumption. In this study, we identified different types of MDMA users and showed that differences in the intentions with which these individuals consume MDMA are associated with different self-reported long-term psychological benefits. While a great deal still needs to be learned about typical patterns of recreational consumption of MDMA—and its long-term impacts—this study provides further evidence highlighting the importance of intentions.

## Methods

All materials, data, and analysis script are available on OSF (https://osf.io/t7p59). More details on the data collection can be found in the preprint by Elsey et al.^30^.

### Participants

Participants were recruited through various online communities with interest in psychedelics, among students of the University of Amsterdam, and via word of mouth. Students could receive research credits for their participation, while all other participants received 50€ vouchers through random lottery draws. Nine-hundred-twenty-four individuals completed the online survey. Thirty-six participants were removed because they either failed to pass the attention checks (*n* = 25) or indicated using MDMA in therapeutic settings (*n* = 13). Additionally, we removed participants who did not complete all relevant parts of the survey (see Materials for a list of relevant variables;* n* = 120). The final sample consisted of 766 individuals, of which 363 (47.4%) identified as male, 384 (50.1%) as female, and 19 (2.5%) as other or undisclosed. The mean age was 29.4 years (*SD* = 9.14). The majority of participants indicated that they lived in the U.S. (*n* = 249, 32.5%), the Netherlands (*n* = 230, 30.0%), Canada (*n* = 81, 10.6%), or the U.K. (*n* = 68, 8.9%). The remaining participants were from other countries around the globe (*n* = 138, 18.0%).

MDMA was last used within the past 6 months by 500 participants (65.3%), within 6 to 24 months by 161 (19.7%), and more than 24 months ago by 115 (15.0%). Of those who had used MDMA within the past 6 months, the majority indicated using it rarely or occasionally (*n* = 392; 63.4%), whereas the remaining 127 (24.5%) indicated using it regularly, very regularly, or on most days.

### Procedure

Individuals who were at least 18 years old and fluent in either English or Dutch were eligible to participate in this study. Upon checking these inclusion criteria and signing the informed consent form, which all participants did, participants provided demographic information. They were then asked a set of items pertaining to their past use of MDMA, including intentions for using it, as well as immediate and long-term positive and negative experiences. Next, participants responded to questions about the physical and social contexts of their use, as well as on the consumption of MDMA mixed with other drugs within the past 6 months and during their highest period of consumption. The questionnaire ended with an MDMA-specific and a general drug addiction scale^44^. All items used in the survey can be retrieved at OSF https://osf.io/t7p59. Items relevant to this analysis are described in this manuscript and in the Supplementary Materials Tables S1, S2. The data collection and survey materials were approved by the University of Amsterdam institutional ethics review board (2020-COP-11936). All methods were carried out in accordance with the guidelines provided by the ethics review board of the University of Amsterdam.

### Materials

The following blocks of survey items were used in the subsequent analyses.

#### Physical and social setting

The physical settings in which MDMA had been consumed was measured with a relative percentage score assigned to seven physical settings [1 = At clubs or raves, 2 = At festivals, 3 = At pubs or bars, 4 = At home (not as part of a house party), 5 = When out in nature (not at an outdoor festival / rave), 6 = At house parties or similar social gatherings, 7 = Other situation (please specify)]. Participants were instructed to assign a percentage to each physical setting in which MDMA has been used in the past. The percentages had to sum up to 100%.

Relative percentage assignments were also used to measure the social settings in which MDMA had been consumed. There were six social settings, which included 1 = Just with my partner/a date (e.g. when at home with your partner), 2 = With friends/people I know, at a place where there are many other people (e.g. going with friends to a club), 3 = Just with group of friends/people I know (e.g. taking it with a group of friends in a private place, or somewhere you won’t be interacting much with people you don’t already know), 4 = By myself at a place where there are many other people (e.g. going to an event without friends to meet new people or to dance by yourself, 5 = By myself (e.g. taking the drug at home, or when out in nature alone), and 6 = Other situation (please specify).

#### Intention

Intentions were measured with eight items, rated on a scale from 0 (Not at all a motivation for me) to 100 (A very strong motivation for me). These eight items represent the following constructs: Energy, Euphoria, Insight, Sociability, Flirtatious, Sex, Coping, and Fit In. Table S1 in the Supplementary Materials shows the precise formulation of these items. The eight response options for intentions were derived from prior research exploring reported motivations for MDMA usage c.f.^7,30, 45^.

#### Long-term benefits

Positive long-term effects of recreational MDMA use were measured with 12 items describing different social and emotional aspects. For each item, participants were asked how the drug may have affected [them] more generally over the long term, at times when [they] are not on the drug or recovering from taking the drug. Each of the 12 items represented one construct: Positive experience, positive memories, aesthetic experiences, friendship quality, social confidence, positivity, letting go, compassion, reflecting emotions, deeper emotions, positive world view, and less problematic drug use. An example item – representing friendship quality – is: Ecstasy/MDMA has helped me develop new or deeper long-term friendships with people. All items were measured on a scale from 0 (Not at all true) to 100 (Completely true). Table S2 in Supplementary Materials shows the precise wording of these items. The set of long-term effects variables – benefits and harms – was drawn from diverse sources c.f.^30^, including reported therapeutic outcomes effects of classic psychedelic drugs, extrapolations from acute effects studies prior MDMA research, anecdotal reports, and other psychologically relevant factors^7,9, 11, 15, 46–50^.

#### Long-term harms

Negative long-term effects of recreational MDMA use were also measured with 12 items. These items partially mirror the items representing the positive long-term outcomes. For example, the Worsened Friendship Quality construct is measured with the item “My long-term friendships have been ruined or worsened by my use of ecstasy/MDMA”, mirroring the friendship item shown above. The items on the long-term harm items are represented by the following constructs: Worst Experience, Troubled Memories, Dull Aesthetic Experiences, Decreased Friendships Quality, Social Anxiety, Hopelessness, Paranoia, Concentration, Worse Emotional Reflection, Shallow Emotions, Negative World View, and Problematic Drug Use. Each item was measured on a scale from 0 (Not at all true) to 100 (Completely true). Table S2 in Supplementary Materials shows the precise wording of these items.

#### Control variables

In our linear regression analyses for RQ3 (see Analytical Strategy section for details), we included a number of control variables. Specifically, we controlled for the effects of gender, age, the frequency of MDMA use within the past 6 months, the number of months since the last MDMA use (with a maximum value of 24), MDMA addiction, and whether the participant reported having been diagnosed with a mental health problem.

The frequency of MDMA use within the past 6 months consisted of five levels [1 = rarely (once in the past 6 months), 2 = occasionally (about two or three times in the past 6 months), 3 = regularly (about once or twice a month in the past 6 months), 4 = very regularly (about once a week in the past 6 months), 5 = most days (used the drug more days than not in the past 6 months)].

MDMA addiction was measured with an adapted version of the CAGE-AID questionnaire^44^ modified for MDMA use. The CAGE-AID was originally designed to evaluate indicators of addiction to alcohol or other substances. It consists of four questions that are answered using a Yes/No format. These questions pertain to attempts to reduce use, criticism received for one's use, feelings of guilt related to use, and using drugs/alcohol first thing in the morning. In the case of the MDMA CAGE, the question about using drugs first thing in the morning was replaced with one that asks about the respondent’s history of bingeing on MDMA, specifically taking the drug repeatedly over a 48-h period without sleeping. For further details on the MDMA-adapted CAGE questionnaire, see Ref.^30^. See Table S7 in the Supplementary Materials for further details on the distribution of these control variables across the clusters.

#### Analytical strategy

In this section, we describe the analytic strategy for each of the three research questions.

##### RQ1: Which types of recreational MDMA users exist with regards to the settings in which they experience MDMA?

We used a clustering algorithm to detect distinct types of MDMA users based on the physical and social setting variables. This analysis was conducted in three steps according to common guidelines for performing a clustering analysis^51,52^. First, we conducted a principal component analysis (PCA) on the 13 variables (seven physical setting variables and six social setting variables, excluding the Other category) to reduce the noise in the data and improve the performance of the clustering algorithm^52^. We used a fuzzy coding approach for the PCA^53^, which is applicable to percentage-type data. Components with eigenvalues greater than one are considered unique components for subsequent analyses. In the second step, we determined the optimal number of clusters (*k*) by computing the average silhouette width *S* for various *k* values and selecting the *k* with the highest average silhouette width *S*. In the final step, we used a K-medoids clustering algorithm (Kaufman & Rousseeuw^31^) to identify distinct types of MDMA consumers based on the components determined by the PCA. For this, we used the *pam* function from the *cluster* R-package (Version: 2.1.4), which uses the original Partition Around Medoids (PAM) algorithm to perform K-medoids clustering^54^. We applied this K-medoids algorithm^31^, instead of the frequently used k-means algorithm, as it provided a more robust result and is less affected by outliers^55^. Using this procedure, each participant was assigned a number corresponding to their type of MDMA use with regards to the setting in which it was consumed. The performance of this procedure was determined by the level of the average silhouette width *S* of the chosen k and by visual inspections of the type-assignment with the initial items.

##### RQ2: Which types of recreational MDMA users exist with regards to the intentions with which they consume MDMA?

The procedure of RQ1 was repeated to assess RQ2. The only difference was that a non-fuzzy coding approach was used, as the intentions variables consisted of independent rating scales, instead of relative percentages.

##### RQ3: are there differences in the perceived long-term social-emotional effects of MDMA use between the identified types of MDMA consumers (in terms of settings and intentions)?

To answer this research question, we estimated a series of multivariable linear regression models. We treated each long-term outcome variable as a separate dependent variable. Independent variables were categorized into three groups: (1) Type of MDMA user based on setting, as identified in RQ1 (dummy coded variables), (2) type of MDMA user based on intention, as identified in RQ2 (dummy coded variables), and (3) control variables, as described above. In addition, for each of the dependent variables, we estimated a model without the variables indicating setting and intention type (while controlling for all other covariates). This approach allowed us to evaluate the contribution of these variables to the explained variance *R*^2^ of the models.

To address the potential for Type I errors due to the large number of statistical tests conducted, we applied a Benjamini–Hochberg correction to the p-value for the within-research-question related tests^56^.

### Supplementary Information

Supplementary Information.

## Data Availability

All materials, data, and analysis script are available on OSF (https://osf.io/t7p59).

## References

[1] European Monitoring Centre for Drugs and Drug Addiction (2016). European Drug Report 2016: Trends and Developments.

[2] Bedi G, Phan KL, Angstadt M, de Wit H (2009). Effects of MDMA on sociability and neural response to social threat and social reward. Psychopharmacology.

[3] Holland J (2001). Ecstasy: The Complete Guide: A Comprehensive Look at the Risks and Benefits of MDMA.

[4] Kelly BC, Parsons JT, Wells BE (2006). Prevalence and predictors of club drug use among club-going young adults in New York city. J. Urban Health Bull. New York Acad. Med..

[5] Regan A, Margolis S, de Wit H, Lyubomirsky S (2021). Does ±3,4-methylenedioxymethamphetamine (ecstasy) induce subjective feelings of social connection in humans? A multilevel meta-analysis. PLoS One.

[6] Rodgers J (2006). Differential experiences of the psychobiological sequelae of ecstasy use: Quantitative and qualitative data from an internet study. J. Psychopharmacol..

[7] Sumnall HR, Cole JC, Jerome L (2006). The varieties of ecstatic experience: An exploration of the subjective experiences of ecstasy. J. Psychopharmacol..

[8] Morgan MJ (2000). Ecstasy (MDMA): A review of its possible persistent psychological effects. Psychopharmacology.

[9] Parrott AC (2013). Human psychobiology of MDMA or ‘Ecstasy’: An overview of 25 years of empirical research. Hum. Psychopharmacol. Clin. Exp..

[10] Rendell PG, Gray TJ, Henry JD, Tolan A (2007). Prospective memory impairment in “ecstasy” (MDMA) users. Psychopharmacology.

[11] Verheyden SL, Henry JA, Curran HV (2003). Acute, sub-acute and long-term subjective consequences of ‘ecstasy’ (MDMA) consumption in 430 regular users. Hum. Psychopharmacol. Clin. Exp..

[12] Carhart-Harris RL, Goodwin GM (2017). The therapeutic potential of psychedelic drugs: Past, present, and future. Neuropsychopharmacology.

[13] Greer GR, Tolbert R, Peroutka SJ (1990). The therapeutic use of MDMA. Ecstasy: The Clinical, Pharmacological and Neurotoxicological Effects of the Drug MDMA.

[14] Parrott AC (2007). The psychotherapeutic potential of MDMA (3,4-methylenedioxymethamphetamine): An evidence-based review. Psychopharmacology.

[15] Carhart-Harris RL, Nutt DJ (2010). User perceptions of the benefits and harms of hallucinogenic drug use: A web-based questionnaire study. J. Subst. Use.

[16] Colbert R, Hughes S (2022). Evenings with Molly: Adult couples’ use of MDMA for relationship enhancement. Cult. Med. Psychiatry.

[17] Johnstad PG (2021). Who is the typical psychedelics user? Methodological challenges for research in psychedelics use and its consequences. Nord. Stud. Alcohol Drugs.

[18] Hartogsohn I (2017). Constructing drug effects: A history of set and setting. Drug Sci. Policy Law.

[19] Leary T, Litwin GH, Metzner R (1963). Reactions to psilocybin administered in a supportive environment. J. Nerv. Ment. Dis..

[20] Liechti ME, Gamma A, Vollenweider FX (2001). Gender differences in the subjective effects of MDMA. Psychopharmacology (Berl.).

[21] Pardo-Lozano R (2012). Clinical pharmacology of 3,4-methylenedioxymethamphetamine (MDMA, ‘ecstasy’): The influence of gender and genetics (CYP2D6, COMT, 5-HTT). PLoS One.

[22] Simmler LD, Liechti ME, Maurer HH, Brandt SD (2018). Pharmacology of MDMA- and amphetamine-like new psychoactive substances. New Psychoactive Substances.

[23] Studerus E, Vizeli P, Harder S, Ley L, Liechti ME (2021). Prediction of MDMA response in healthy humans: A pooled analysis of placebo-controlled studies. J. Psychopharmacol..

[24] Bedi G (2011). Individual differences in acute responses to MDMA in humans: Effects of sex and past ecstasy use. TOADDJ.

[25] Kirkpatrick MG (2014). MDMA effects consistent across laboratories. Psychopharmacology (Berl.).

[26] Kirkpatrick MG, de Wit H (2015). MDMA: A social drug in a social context. Psychopharmacology.

[27] Boeri MW, Sterk CE, Elifson KW (2004). Rolling beyond raves: Ecstasy use outside the rave setting. J. Drug Issues.

[28] Solowij N, Hall W, Lee N (1992). Recreational MDMA use in Sydney: A profile of ‘Ecstasy’ users and their experiences with the drug. Br. J. Addict..

[29] Watson L, Beck J (1991). New age seekers: MDMA use as an adjunct to spiritual pursuit. J. Psychoact. Drugs.

[30] Elsey, J., Wuestman, V. A. F. & Fieten, A. *User perceptions of long-term costs and benefits of MDMA use: Findings from a large online sample*. https://osf.io/wqbmg, 10.31234/osf.io/wqbmg (2021).

[31] Kaufman L, Rousseeuw PJ (2009). Finding Groups in Data: An Introduction to Cluster Analysis.

[32] Cohen J (1988). Statistical Power Analysis for the Behavioral Sciences.

[33] Davison D, Parrott AC (1997). Ecstasy (MDMA) in recreational users: Self-reported psychological and physiological effects. Hum. Psychopharmacol. Clin. Exp..

[34] Lyubomirsky S (2022). Toward a new science of psychedelic social psychology: The effects of MDMA (Ecstasy) on social connection. Perspect. Psychol. Sci..

[35] Wolff M (2020). Learning to let go: A cognitive-behavioral model of how psychedelic therapy promotes acceptance. Front. Psychiatry.

[36] Mitchell JM (2021). MDMA-assisted therapy for severe PTSD: A randomized, double-blind, placebo-controlled phase 3 study. Nat. Med..

[37] Mullard A (2021). MDMA scores PTSD success in a landmark phase III trial. Nat. Rev. Drug Discov..

[38] Yudkin DA (2022). Prosocial correlates of transformative experiences at secular multi-day mass gatherings. Nat. Commun..

[39] Wolff M, Mertens LJ, Walter M, Enge S, Evens R (2022). The acceptance/avoidance-promoting experiences questionnaire (APEQ): A theory-based approach to psychedelic drugs’ effects on psychological flexibility. J. Psychopharmacol..

[40] Johnstad, P. Entheogenic spirituality: Exploring spiritually motivated entheogen use among modern Westerners. *JEQR ***12**, 244–260 (2018).

[41] Haijen ECHM (2018). Predicting responses to psychedelics: A prospective study. Front. Pharmacol..

[42] Griffiths RR, Richards W, Johnson M, McCann U, Jesse R (2008). Mystical-type experiences occasioned by psilocybin mediate the attribution of personal meaning and spiritual significance 14 months later. J. Psychopharmacol..

[43] Griffiths RR (2011). Psilocybin occasioned mystical-type experiences: Immediate and persisting dose-related effects. Psychopharmacology.

[44] Brown, R. L. & Rounds, L. A. Conjoint screening questionnaires for alcohol and other drug abuse: Criterion validity in a primary care practice. *Wis. Med. J.* **94**(3), 135–140 (1995). 7778330

[45] Ter Bogt TFM, Engels RCME (2005). “Partying” hard: Party style, motives for and effects of MDMA use at rave parties. Subst. Use Misuse.

[46] Mithoefer MC (2019). MDMA-assisted psychotherapy for treatment of PTSD: Study design and rationale for phase 3 trials based on pooled analysis of six phase 2 randomized controlled trials. Psychopharmacology (Berl.).

[47] Wagner MT (2017). Therapeutic effect of increased openness: Investigating mechanism of action in MDMA-assisted psychotherapy. J. Psychopharmacol..

[48] Bershad AK, Miller MA, Baggott MJ, de Wit H (2016). The effects of MDMA on socio-emotional processing: Does MDMA differ from other stimulants?. J. Psychopharmacol..

[49] Kamilar-Britt P, Bedi G (2015). The prosocial effects of 3,4-methylenedioxymethamphetamine (MDMA): Controlled studies in humans and laboratory animals. Neurosci. Biobehav. Rev..

[50] Elsey JWB (2017). Psychedelic drug use in healthy individuals: A review of benefits, costs, and implications for drug policy. Drug Sci. Policy Law.

[51] Bécue-Bertaut M, Pagès J (2008). Multiple factor analysis and clustering of a mixture of quantitative, categorical and frequency data. Comput. Stat. Data Anal..

[52] Xu Q, Ding C, Liu J, Luo B (2015). PCA-guided search for K-means. Pattern Recognit. Lett..

[53] Chevene F, Doléadec S, Chessel D (1994). A fuzzy coding approach for the analysis of long-term ecological data. Freshw. Biol..

[54] Schubert E, Rousseeuw PJ (2021). Fast and eager k-medoids clustering: O(k) runtime improvement of the PAM, CLARA, and CLARANS algorithms. Inf. Syst..

[55] Arora P, Deepali, Varshney S (2016). Analysis of K-means and K-medoids algorithm for big data. Procedia Comput. Sci..

[56] Benjamini Y, Hochberg Y (1995). Controlling the false discovery rate: A practical and powerful approach to multiple testing. J. R. Stat. Soc. Ser. B (Methodol.).

