# Supplementary material for: Subjective long-term emotional and social effects of recreational MDMA use: the role of setting and intentions
Source: Sci Rep. 2024 Feb 10;14:3434. doi: 10.1038/s41598-024-51355-6 (PMC10858867; doi:10.1038/s41598-024-51355-6)
Supplement: Supplementary file 1 — Supplementary Information. [file 41598_2024_51355_MOESM1_ESM.pdf]

Supplementary Materials

Subjective Long-Term Emotional and Social Effects of Recreational MDMA Use: The Role of Setting and Intentions

Timon Elmer[1*], Tanya K.Vannoy[2], Erich Studerus[3], and Sonja Lyubomirsky[2]

[1]Department of Psychology, University of Zurich, Zurich, Switzerland

[2]Department of Psychology, University of California, Riverside, USA

[3]Department of Psychology, University of Basel, Basel, Switzerland

Table S1

Intention Items

Construct	Item
Energy	To give me energy (e.g., so I don't get tired, to have more energy, so I can dance all night)
Euphoria	To feel euphoric (e.g., so I can feel amazing, to be euphoric, to totally get lost in the music)
Insight	To get better insight into myself (e.g., to get to know myself better, to broaden my consciousness)
Sociability	To be more sociable and open (e.g., to connect with people more, to be more open with people, to ease talking with others)
Flirtatious	To be more flirtatious (e.g., to make flirting easier, to make it easier to 'hit on' someone, to be more open to being approached)
Sex	To enhance sex (e.g., to make sex feel better)
Coping	To help deal with or forget my problems (e.g., to have less worries, to feel good for once, to forget my problems)
Fit In	To fit in or go along with other people (e.g., because all my friends do it, my friends push me, it's not fun being sober when no one else is, to fit in with others)

Table S2

Perceived Long-Term Social-Emotional Benefits and Harms of MDMA Use Items

Construct	Item
Perceived Benefits	
Best Experience Ever	My best experiences on ecstasy/MDMA have been among the most positive experiences I've ever had
Amazing Memories	My use of ecstasy/MDMA has provided me with amazing memories that I enjoy looking back on
Appreciate Aesthetic Experiences	I feel like my use of ecstasy/MDMA has somehow enabled me to appreciate more deeply or intensely some aesthetic experiences, such as listening to music or being in nature – even when I am not on the drug
Improved Friendship Quality	Ecstasy/MDMA has helped me develop new or deeper long-term friendships with people
Increased Social Confidence	My experiences with ecstasy/MDMA have made me feel more socially confident or outgoing in daily life
Increased Positivity	I believe ecstasy/MDMA use has, at times, increased my feelings of positivity or hope about life and the future – in a way that has extended beyond just an 'after-glow' period in the days after taking it

Increased Authenticity	Using ecstasy/MDMA has helped me realise I can let go or express myself and nothing bad will happen, and that has had a positive effect on my life
Increased Compassion	My experience of using ecstasy/MDMA has caused me to become a more compassionate person
Better Emotion Reflection	Because of using ecstasy/MDMA, I have somehow become *more* willing or able to acknowledge and reflect on my feelings and emotions
Deep Emotion Experience	My use of ecstasy/MDMA has enabled me to have richer and deeper emotional experiences in my daily life, even when not on the drug
Positive World View	Because of using ecstasy/MDMA, I find I am better able to see the world as a positive and welcoming place
Reduced Other Drug Use	Because of taking ecstasy/MDMA, I stopped or reduced my use of some other drugs that I think might be problematic

Perceived Harms

Worst Experience Ever	My worst experiences on ecstasy/MDMA have been among the most unpleasant experiences I’ve ever had

Unpleasant Memories	My use of ecstasy/MDMA has caused me to have unpleasant memories that trouble me
Dampen Aesthetic Experiences	I feel like my use of ecstasy/MDMA has somehow dulled or dampened my ability to appreciate some aesthetic experiences, such as listening to music or being in nature – even when I am not on the drug
Worsened Friendship Quality	My long-term friendships have been ruined or worsened by my use of ecstasy/MDMA
Increased Social Anxiety	My experiences with ecstasy/MDMA have made me feel more socially anxious or inhibited in daily life
Decreased Positivity	I believe ecstasy/MDMA use has, at times, worsened my feelings of depression or hopelessness about life and the future – in a way that has extended beyond just a 'come-down' period in the days after taking it
Increased Paranoia	I've become more paranoid in daily life because of using ecstasy/MDMA – like sensing people are looking at me or talking about me when they probably are not
Issues Concentrating	My use of ecstasy/MDMA has caused me to have problems with my concentration or memory – I can't focus on things or remember things so well anymore because of my use

Worse Emotion Reflection	Because of using ecstasy/MDMA, I have somehow become less willing or able to acknowledge and reflect on my emotions and feelings
Shallow Emotion Experience	My use of ecstasy/MDMA has caused me to have more blunted and shallower emotional experiences in my daily life, even when not on the drug
Negative World View	Because of using ecstasy/MDMA, I find I am more inclined to see the world as a negative and unfriendly place
Increased Other Drug Use	Because of taking ecstasy/MDMA, I developed issues with some other drugs that I think might be problematic

Table S3

Means and Standard Deviations of Setting and Intention Variables

Type	Variable	*Mean*	*SD*
Physical Setting	Club/Rave	28.24	26.18
	Festival	31.04	29.54
	Pub/Bar	2.33	5.75
	Home	17.55	27.64
	Nature	5.28	12.73
	House Party	14.35	19.09
Social Setting	Partner	12.64	23.63
	Friends (Public)	54.43	36.07
	Friends (Private)	23.33	26.71
	Self (Public)	3.52	12.79
	Self (Private)	4.97	14.54
Intention	Energy	45.06	35.68
	Euphoria	82.51	25.40
	Insight	52.05	38.74
	Sociability	54.84	35.49
	Flirtatious	16.87	26.42
	Sex	19.22	28.30
	Coping	25.13	32.19
	Fit In	11.68	22.20

Note. *N* = 766. *SD* = Standard Deviation.

Table S4

Means and Standard Deviations of Long-Term Benefits and Harms

Type	Variable	*Mean*	*SD*
Benefits	Best Experience Ever	73.06	28.70
	Amazing Memories	86.00	22.14
	Appreciate Aesthetic Experiences	60.58	34.19
	Improved Friendship Quality	61.26	35.02
	Increased Social Confidence	46.53	34.09
	Increased Positivity	53.62	37.28
	Increased Authenticity	57.63	34.44
	Increased Compassion	50.93	36.58
	Better Emotion Reflection	54.00	35.85
	Deep Emotion Experience	51.07	36.48
	Positive World View	50.10	34.02
	Reduced Other Drug Use	17.91	29.74
Harms	Worst Experience Ever	11.45	23.50
	Unpleasant Memories	9.43	19.97
	Dampen Aesthetic Experiences	7.62	18.37
	Worsened Friendship Quality	4.31	13.65

Increased Social Anxiety	7.53	18.38
Decreased Positivity	16.30	28.49
Increased Paranoia	5.90	15.95
Issues Concentrating	15.21	24.53
Worse Emotion Reflection	5.52	15.53
Shallow Emotion Experience	7.73	17.34
Negative World View	4.72	13.48
Increased Other Drug Use	7.87	19.20

Note. *N* = 766. *SD* = Standard Deviation.

Table S5

Correlations Among Setting Variables

		1	2	3	4	5	6	7	8	9	10
Physical Setting	Club/Rave										
	Festival	-0.25***									
	Pub/Bar	0.02	-0.14***								
	Home	-0.4***	-0.44***	-0.11**							
	Nature	-0.21***	-0.25***	-0.03	0.01						
	House Party	-0.22***	-0.31***	0.08*	-0.16***	0.01					
Social Setting	Partner	-0.26***	-0.3***	-0.07*	0.62***	0.13***	-0.11**				
	Friends (Public)	0.45***	0.48***	0.01	-0.63***	-0.23***	-0.25***	-0.51***			
	Friends (Private)	-0.27***	-0.27***	0.04	0.10**	0.17***	0.47***	-0.15***	-0.55***		
	Self (Public)	-0.02	0.08*	0.05	-0.07	-0.03	0.02	-0.07	-0.23***	-0.11**	
	Self (Private)	-0.16***	-0.25***	-0.01	0.43***	0.07*	-0.05	0.00	-0.34***	-0.09*	0.02

Table S6

Correlations Among Intention *Variables*

	1	2	3	4	5	6	7
Energy	-						
Euphoria	0.37***	-					
Insight	-0.20***	-0.15***	-				
Sociability	0.18***	0.24***	0.28***	-			
Flirtatious	0.22***	0.17***	0.08*	0.38***	-		
Sex	0.08*	0.13***	0.2***	0.18***	0.36***	-	
Coping	0.15***	0.16***	0.13***	0.26***	0.24***	0.17***	-
Fit In	0.2***	0.1**	-0.08*	0.17***	0.23***	0.1**	0.23***

Robustness Analyses

We conducted several analyses to test the robustness of our results under different assumptions and procedures. Specifically, (a) we assessed a two-cluster solution for the setting and intention variables, (b) we applied the clustering algorithms without first performing a PCA, (c) we analyzed RQ3 with the principal components as independent variables instead of the clusters identified by the clustering algorithm, and (d) we ran a regression analysis without any covariates (i.e., only the dummy variables for the intention and setting clusters).

For the two-cluster solution, we identified a Party Setting With Friends (n = 406) and a Mixed Physical and Social Settings (n = 360) cluster for the setting variables, and a Euphoria and Energy (n = 336) and a Mixed Intentions (n = 430) cluster for the intention variables. Figures S1 and S2 show how these clusters correspond with the item averages of the setting and intention variables, respectively. The results for RQ3, as displayed in Figure S3, reveal no differences between the two setting clusters. Figure S3 also shows higher values in the Mixed Intentions cluster, compared to the Euphoria and Energy cluster, for all the long-term benefit variables, but no differences for the long-term harms.

Even when we did not perform a PCA prior to applying the clustering algorithms, a two-cluster solution still emerged (setting: n_1 = 402; n_2 = 364; intention: n_1 = 342, n_2 = 424), which we do not report in detail here due to its similarity to the two-factor solution described above. The R-Markdown analysis script available on OSF describes these results in detail. Figure S4 in the Supplementary Materials shows the RQ3 results of the analyses without PCA prior to clustering. Results are similar to the two-cluster solution described in Figure S3. The RQ3 analysis using the obtained principal component scores directly in the regression analyses, without the cluster

component, are reported in Figure S5. These results indicate that the setting components are not associated with long-term outcomes, while both principal components of the intention variables are related to long term outcomes.

A regression analysis without controlling for any of the covariates (i.e., the model only consisted of the dummy variables for setting and intention clusters) is presented in Figure S6. While the conclusions about the long-term benefits remain identical, some of the intention effects on long-term harms become significant when leaving covariates out of the model. Specifically, the Mixed Intentions clusters showed slightly higher values in long-term harms than the reference category (Euphoria and Energy). For detailed results and an analysis on differences between covariates and the identified clusters, see Figure S6 and Tables S7-S8. Overall, these robustness analyses align well with the main results described above and lead to similar conclusions.

Figure S1

Two Identified Setting Clusters and Their Average Values per Setting Variable

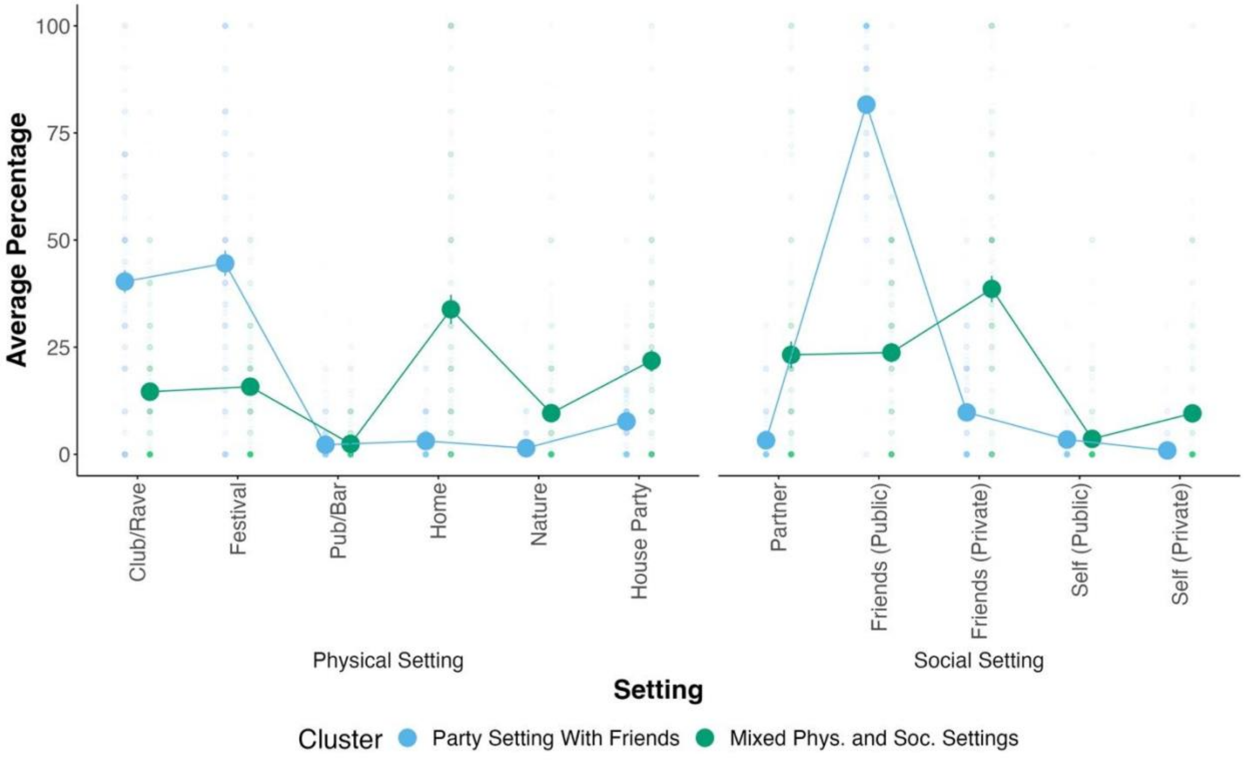

Figure S2

Two Identified Intention Clusters and Their Average Values per Variable

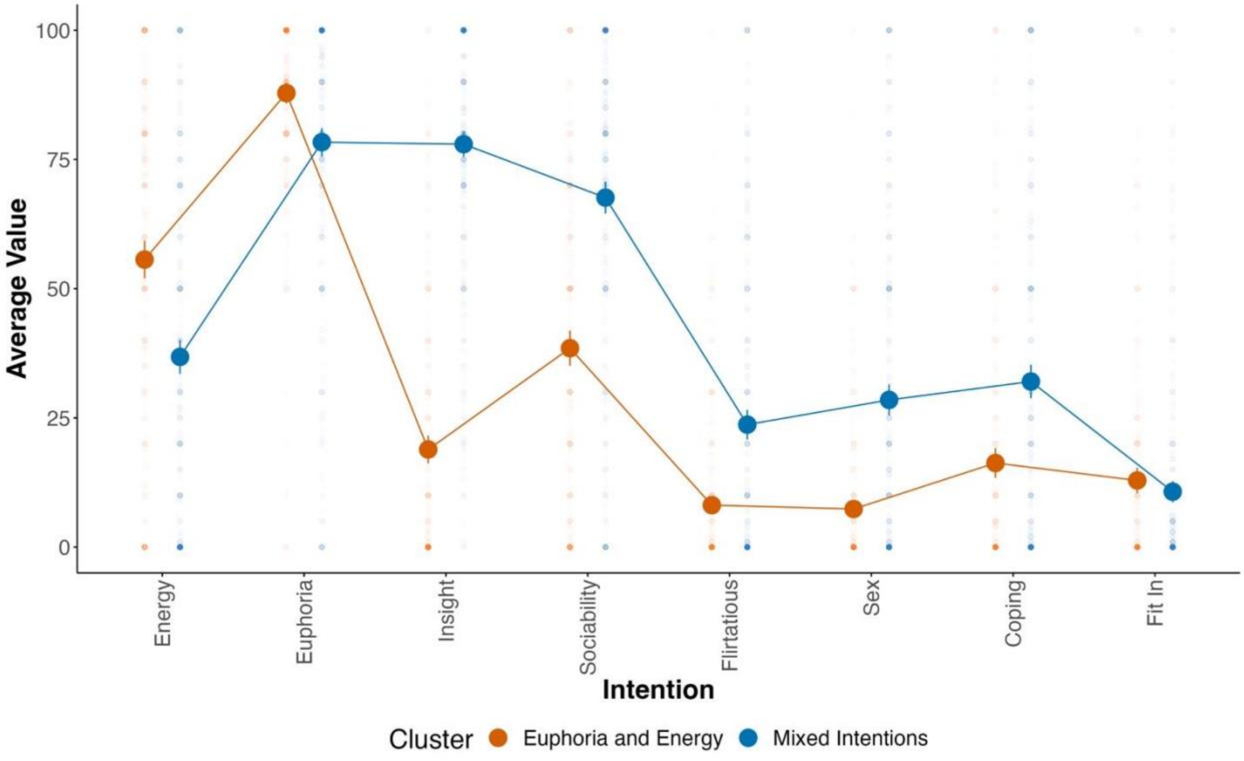

Figure S3

Regression Coefficients of the Setting and Intention Variables per Perceived Long-Term Outcome for Two-Cluster Solutions

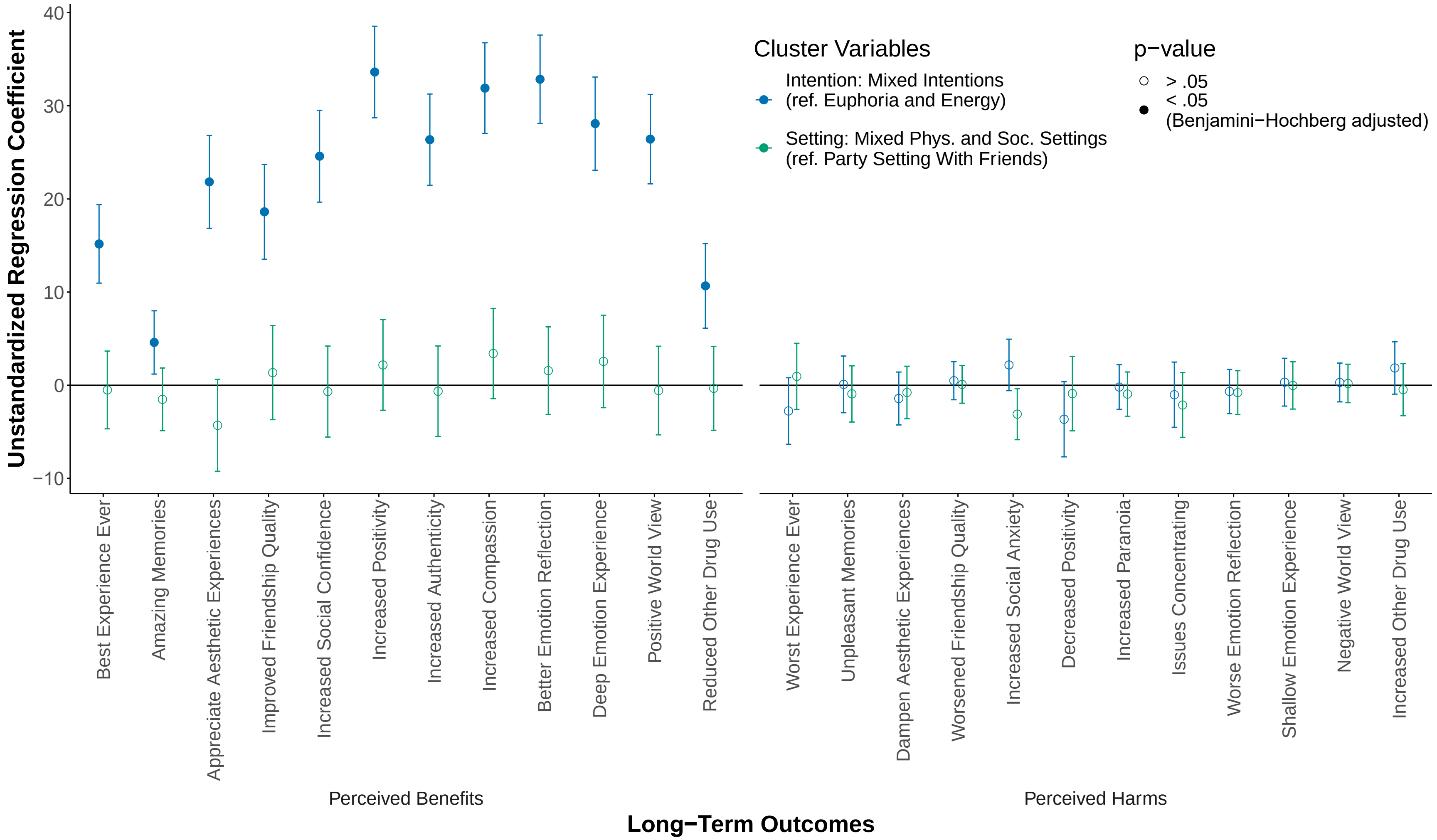

Figure S4

Regression Coefficients of the Setting and Intention Variables per Long-Term Outcome Without PCA Before Clustering

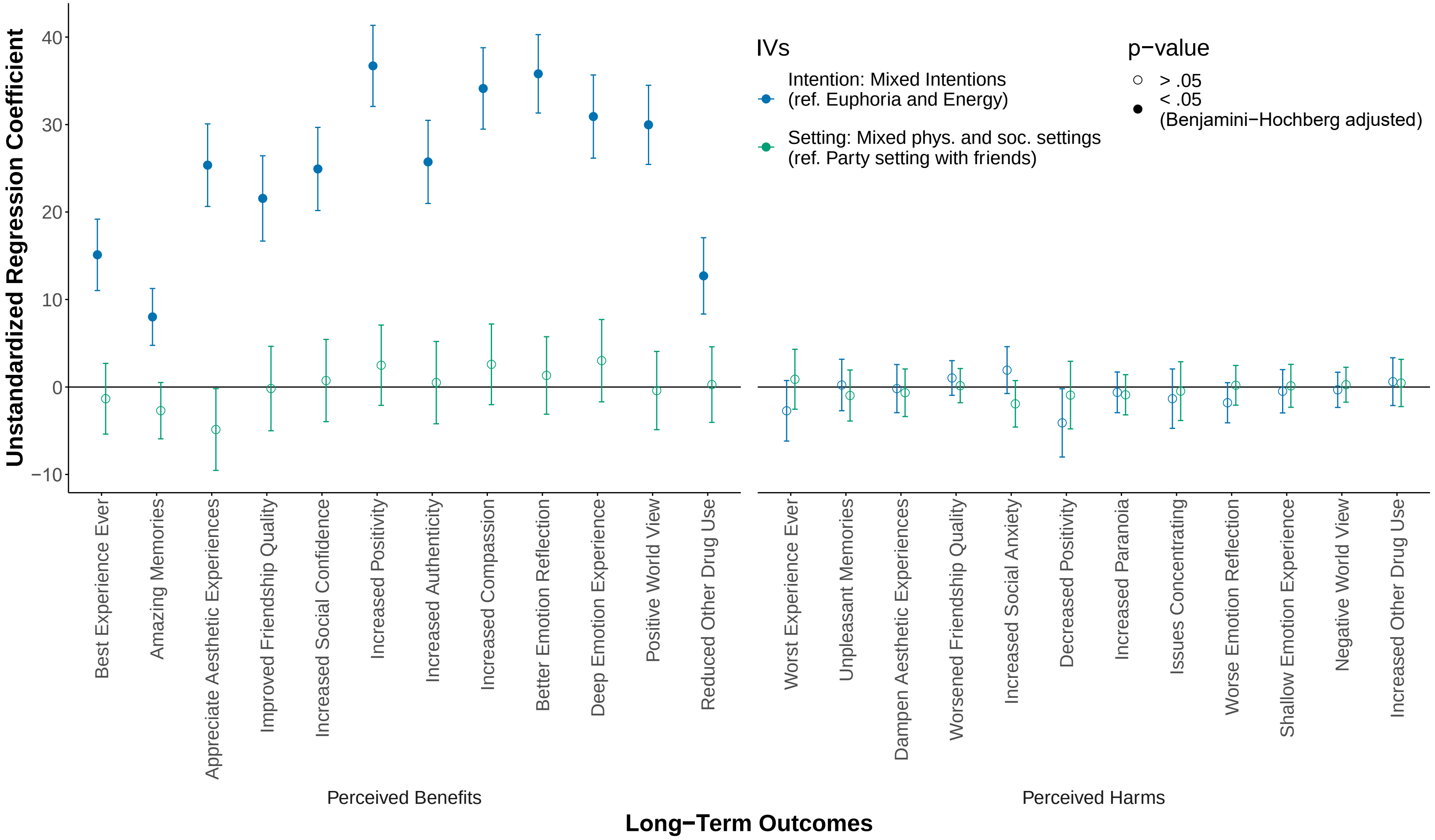

Figure S5

Regression Coefficients with 95% Confidence Intervals per Long-Term Outcome Without Clustering (With Components Used as Independent Variables)

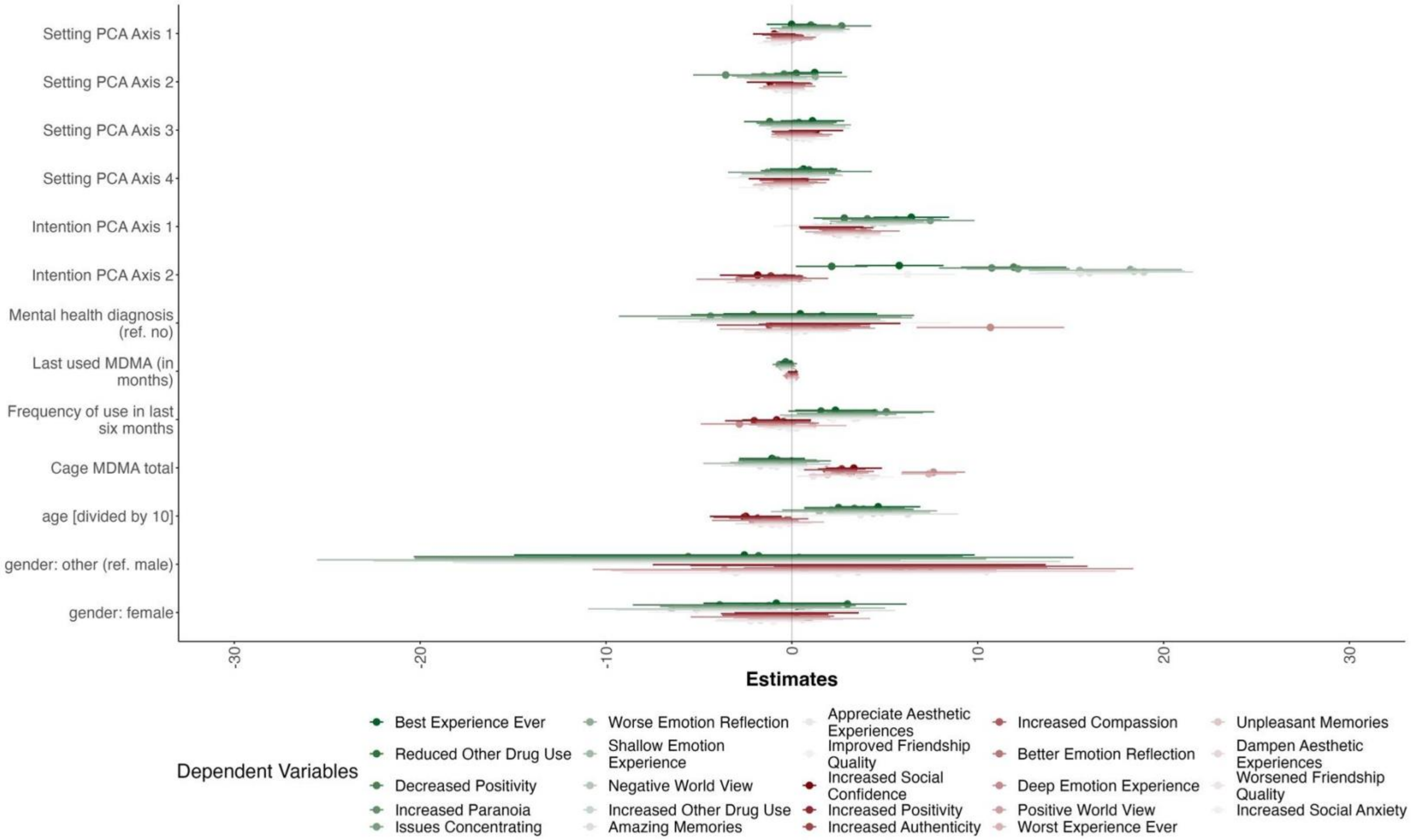

Note. Detailed model results can be obtained from the R-Markdown analysis script available on OSF.

Figure S6

Regression Coefficients of the Setting and Intention Variables per Long-Term Outcome Without Controlling for Any Covariates

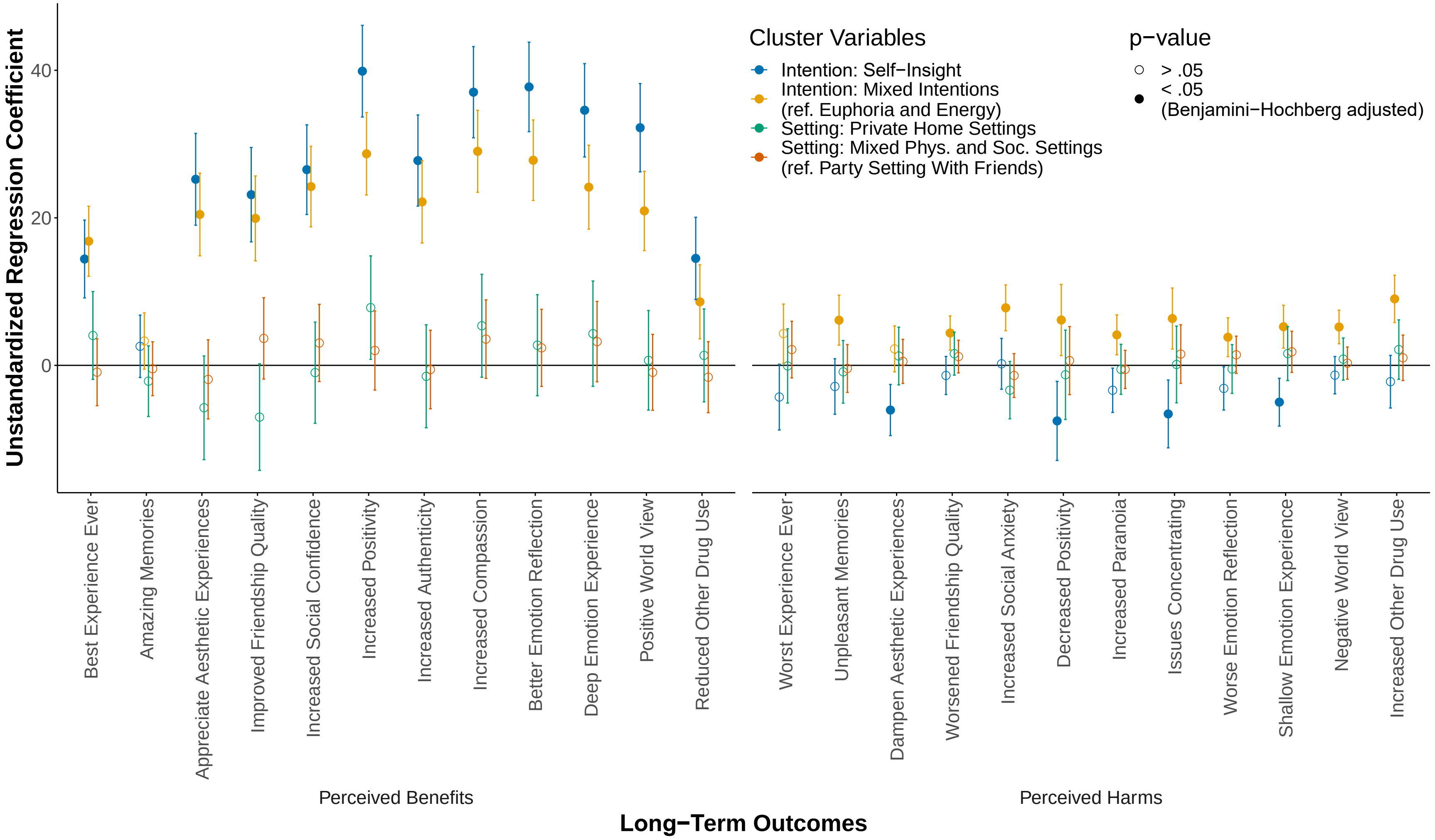

Note. Detailed model results can be obtained from the R-Markdown analysis script available on OSF

Table S7

Differences Between Setting and Intention Clusters by Continuous Covariates

	Settings								Intentions							
	Party Setting With Friends		Private Home Settings		Mixed Settings				Euphoria and Energy		Self-Insight		Mixed Intentions			
	Mean	*SD*	*Mean*	*SD*	*Mean*	*SD*	*F*	*p*	*Mean*	*SD*	*Mean*	*SD*	*Mean*	*SD*	*F*	*p*
Most recent MDMA use [in months]	6.78	7.56	7.01	8.77	7.99	8.45	1.727	.178	6.82	7.62	6.97	8.12	7.91	8.56	2.348	.126
Frequency of MDMA use in past 6 months	1.31	1.13	1.46	1.22	1.29	1.17	1.062	.346	1.33	1.14	1.31	1.11	1.36	1.23	0.064	.801
CAGE MDMA score	1.15	1.18	0.90	1.17	1.35	1.18	6.238	.002	1.15	1.16	0.78	1.02	1.54	1.24	12.677	<.001
Age [in years]	27.34	7.26	34.30	11.37	30.01	9.46	31.592	<.001	27.24	8.06	31.75	10.13	29.95	8.95	13.892	<.001

Note. N = 766, df = 2, M = Mean, SD = Standard Deviation, F = F value of an ANOVA analysis comparing the cluster means.

Differences Between Clusters in Covariates

Table S7 shows the results of a series of ANOVA analyses between the settings (left panel) and the intention (right panel) clusters and the three continuous covariates that were used in the regression models to analyze RQ3. As Table S7 indicates there are significant differences between the intention and setting clusters in the CAGE MDMA score (representing the degree to which participants perceive to be addicted to MDMA) and age. There were also some significant differences between the clusters in the categorical covariates: While the setting clusters differed by gender identity $\chi^2(4) = 13.32, p = .010$, the intention clusters did not $\chi^2(4) = 3.01, p = .556$. There were no differences between the setting clusters in having (ever) had a mental health diagnosis $\chi^2(2) = 4.31, p = .120$, but there were between the intention clusters $\chi^2(4) = 17.26, p =< .001$. Table S8 shows the numbers of individuals per cluster and mental health and gender identification.

Table S8

Cell Sizes of Setting and Intention Clusters by Binary Covariates

	Mental Health Diagnosis		Gender Identification		
	No	Yes	Male	Other/Not Disclosed	Female
Setting Clusters					
Party Setting With Friends	264	124	173	4	211
Private Home Settings	77	55	69	3	60
Mixed Settings	165	81	121	12	113
Intention Clusters					
Euphoria and Energy	226	76	136	5	161
Self-Insight	134	85	107	7	105
Mixed Intentions	146	99	120	7	118
